# *Mycobacterium tuberculosis* P-Type ATPases: Possible Targets for Drug or Vaccine Development

**DOI:** 10.1155/2014/296986

**Published:** 2014-07-10

**Authors:** Lorena Novoa-Aponte, Carlos Yesid Soto Ospina

**Affiliations:** Chemistry Department, Faculty of Sciences, Universidad Nacional de Colombia, Carrera 30 No. 45-03, Bogotá, Cundinamarca 111321, Colombia

## Abstract

Tuberculosis (TB) has been the biggest killer in the human history; currently, *Mycobacterium tuberculosis* (*Mtb*) kills nearly 2 million people each year worldwide. The high prevalence of TB obligates the identification of new therapeutic targets and the development of anti-TB vaccines that can control multidrug resistance and latent TB infections. Membrane proteins have recently been suggested as key targets for bacterial viability. Current studies have shown that mycobacteria P-type ATPases may play critical roles in ion homeostasis and in the response of mycobacteria to toxic substances in the intraphagosomal environment. In this review, we bring together the genomic, transcriptomic, and structural aspects of the P-type ATPases that are relevant during active and latent *Mtb* infections, which can be useful in determining the potential of these ATPases as drug targets and in uncovering their possible roles in the development of new anti-TB attenuated vaccines.

## 1. Introduction

*Mycobacterium tuberculosis *(*Mtb*), the causative agent of tuberculosis (TB), kills approximately one person every 25 seconds which means that 2 million people worldwide die of TB every year [1]. The current high TB prevalence is mainly due to pervasive poverty, coinfection with human immunodeficiency virus (HIV), and the emergence of* Mtb* strains that are resistant to the common drugs used to treat TB (MDR and XDR strains). Each of these factors has positioned this infectious disease as a major public health problem [2]. Controlling TB is complicated due to several factors, of which the most important is the capacity of* Mtb* to persist for long periods of time in adverse conditions into macrophages [3, 4]. In the intraphagosomal environment, mycobacteria face ion and nutrient deficiencies, as well as an arsenal of toxic substances, such as acid hydrolases, antimicrobial peptides, reactive oxygen and nitrogen species, and high concentrations of heavy metal cations, among others [5–11]. The outcome of* Mtb* infection would be represented as a distribution between active TB and latent TB on the basis of the presence or absence of clinical symptoms, respectively, making TB difficult to control and diagnose [12]. Thus, it is essential to identify specific therapeutic targets that can be used to control latent and active TB infections. In this regard, a new anti-TB drug should achieve the following: (i) shorten the duration and dose of treatment; (ii) be active against both MDR and XDR* Mtb* strains; (iii) be able to eradicate latent TB; and (iv) be capable of being coadministered with drugs to treat HIV [13, 14].

The identification of new therapeutic targets in mycobacteria takes into account aspects that allow mycobacteria to adapt and succeed during the infectious process. Based on the current knowledge, P-type ATPases might be important as they transport metal cations across the plasma membranes of both the host and pathogen and generate electrochemical gradients necessary for the transport of other solutes and for the protection of the cell against the toxic substances in the phagosomes [15, 16]. For example, the deletion of the* Mtb* CtpV P-type ATPase reduces the resistance of the tubercle bacilli to the toxic levels of copper ions in its surroundings. This deletion also diminishes the bacteria's ability to grow inside the lungs in murine infection models, suggesting that this pump is required to maintain the virulence of the tubercle bacilli [17].

The aim of this review is to present the potential of* Mtb* P-type ATPases as possible therapeutic targets and to assess their usefulness in the development of vaccines for TB control. This potential will be evaluated based on three different aspects: (i) experimental evidence for differential gene expression patterns of these ion transporters during critical mycobacterial infection processes and treatment with toxic substances; (ii) the existence of close homologs of the* Mtb* P-type ATPases in other pathogens and their considerable divergence with the human P-type transporters; and (iii) the apparent impairment of* Mtb* virulence after deleting the aforementioned ion pumps.

## 2. P-Type ATPases

**Figure 1:**
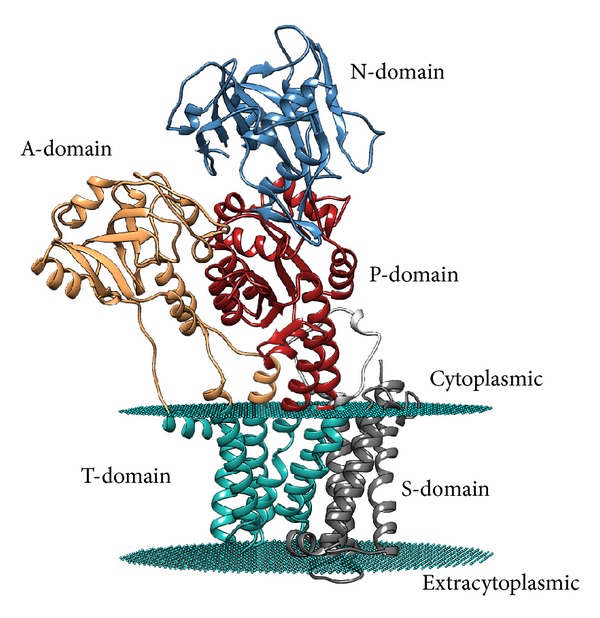
Structural organization of the five P-type ATPase domains.* M. tuberculosis *CtpF tertiary structure model, generated using the SWISS-MODEL server (http://swissmodel.expasy.org/) in automated mode and the structure of the Sarco(endo)plasmic reticulum calcium ATPase 1 from* Oryctolagus cuniculus* (PDB accession number 3AR4) as template. The model was modified using the PPM server to include the possible location of the lipid bilayer, which is displayed in cyan dummies that correspond to the location of the carbonyl groups in the bilayer.

In general, the ATPase enzymes are membrane proteins that perform energetically unfavorable processes, such as folding and degrading proteins, the initiation of replication, DNA repair, and the transport of substances [18], using the energy released during ATP hydrolysis. Specifically, P-type ATPases are active transporters of metal cations that are autophosphorylated on a conserved aspartate residue during each catalytic cycle, and these enzymes can be inhibited by orthovanadate [19]. All P-type ATPases have multiple domains (Figure 1): three cytoplasmic domains (A, actuator; N, nucleotide binding; P, phosphorylation) and two domains embedded in the membrane (T, transport; S, class specific support) [15, 19–21]. During the catalytic cycle, the phosphorylation process is catalyzed by the N domain (protein kinase activity), while dephosphorylation is catalyzed by the A domain (phosphatase activity). The phosphorylation/dephosphorylation cycle is accompanied by conformational changes in the enzyme that allow cation transport, which is mediated by the T domain, which also exhibits amino acid determinants that define the ion specificity of each type of pump [15, 19].

### 2.1. The Tubercle Bacillus Has a Large and Unusual Number of P-Type ATPases

*Mtb *has 11 P-type ATPases (CtpA, CtpB, CtpC, CtpD, CtpE, CtpF, CtpG, CtpH, CtpI, CtpJ, and CtpV), as shown in Table 1. Seven of these enzymes have been identified as possible carriers of heavy metal cations, suggesting their possible role in the intraphagosomal survival of* Mtb* [22]. Metals, such as iron, magnesium, cobalt, copper, manganese, and zinc, are essential for all forms of life, as they act as members of prosthetic groups or as cofactors of many enzymes. In general, microorganisms only need traces of these micronutrients for proper cell function; indeed, excessive accumulation is toxic [23]. An overabundance of metals can block enzymatic functional groups, displace essential metal ions, and modify the active conformations of biomolecules [24]. In response, cells have developed strategies to maintain a balance between the entrance and the exit of cations to preserve their concentrations at nutrient levels. Transport systems involved in cell homeostasis must be pivotal for the virulence of intracellular pathogens, such as* Mtb* [17]. The large number of P-type ATPases encoded in the genome of* Mtb* is twice the size of those encoded in the genomes of saprophyte mycobacteria, such as* M. smegmatis* [25]; this may strongly suggest the importance of this type of metal cation transporters to the virulence of the tubercle bacillus [26, 27].

### 2.2. P-Type ATPases Have Been Associated with the Intracellular Survival of Mycobacteria

Recently, it has been established that the heavy metal P-type ATPases CtpC and CtpD are necessary for murine infection with* Mtb* [28]. In line with this discovery, it has been proposed that the heavy metal pumps CtpC, CtpV, and CtpG are part of a defense mechanism used by mycobacteria to survive for long periods of time within human phagocytic cells [11, 17]. For example, deletion of CtpC, a Zn^2+^ efflux system, in* Mtb* cells causes the tubercle bacilli to be hypersensitive to physiologic Zn^2+^ concentrations, thereby affecting their capacity to grow inside macrophages [11]. In general, it could be suggested that deletion of nutrient uptake systems significantly attenuates the growth of* Mtb* strains [28].

### 2.3. *Mycobacterium tuberculosis* P-Type ATPases Are Located in the Plasma Membrane

In general, membrane proteins are considered key therapeutic targets due to their roles as substance carriers and mediators in the interactions between pathogens and the surrounding environment. Additionally, the P-type ATPases are located in the plasma membrane, which makes them highly accessible [29] to antimicrobial compounds. These properties are advantageous for the development of new drugs because the permeability of this biological barrier does not have to be resolved [14]. Consistent with this assertion, certain anti-TB drugs, such as diarylquinolines (TMC207) and benzothiazines (BTZ043), have been recently developed to target molecules located in the mycobacterial plasma membrane [14]. Therefore,* Mtb* P-type ATPases could be possible targets for the design of inhibitors that are capable of acting on their targets without being internalized by the mycobacterial cell.

While the location of P-type ATPases on the plasma membrane may be advantageous for their accessibility, this location also could be problematic, as it can be difficult to determine the tertiary structure of this kind of proteins which is useful for molecular docking experiments. The appropriate tertiary structure of target proteins is crucial for a rational drug design. An optional strategy may involve the use of the crystal structures of close homologues that share at least 70% identity [30]. Certainly, the constant development of crystallization methods for membrane proteins will make this strategy more feasible in the near future.

## 3. P-Type ATPases Are Useful Drug Targets

For the development of rational drugs against specific cellular targets, information about the chemical structure of similar inhibitors is pivotal for the design of new molecules with improved affinities and diminished minimal inhibitory concentrations. For example, the anti-TB drug rifabutin is a rifamycin derivative that was developed to replace rifampicin in the treatment of HIV-TB coinfections [14]. On the other hand, P-type ATPases are the general targets of many drugs [18, 29]. For example, omeprazole, which is used to treat gastric ulcers, acts as an inhibitor of an H^+^/K^+^ ATPase, and thapsigargin, a Ca^2+^ SERCA ATPase inhibitor, is used as a prodrug for prostate cancer treatment [29]. Interestingly, inhibitors of P-type ATPases have also been developed as antibiotics; for example, clotrimazole, an antifungal that blocks certain Ca^2+^ ATPase, and chloroquine/artemisinin, which inhibit* Plasmodium falciparum *P-type ATPases, have been used as antimalarial drugs [29].

## 4. Potential of P-Type ATPases as Targets for the Development of Novel Antituberculous Drugs

Currently, the exploration for new anti-TB drugs is based, in part, on* Mtb* gene expression profile data from different stress conditions [4, 30]. In this review, the potential of* Mtb* P-type ATPases as therapeutic targets is specifically considered according to the following: (i) the gene expression profiles of the P-type ATPases in* Mtb* facing toxic substances and under both latent infection and active disease; (ii) their roles in virulence; and (iii) their structural divergences from human P-type ATPases.

## 5. Transcriptional Behavior of* Mycobacterium tuberculosis* P-Type ATPases under Stress Conditions

The transcriptional behavior of P-type ATPases in the presence of toxic substances and in models of latent infection and active disease could suggest strategies used by* Mtb* to colonize, interact with, and/or alter the functions of the host cells [31]. A detailed review of the published* Mtb* gene expression profiles (Table 2) shows the activation or repression of most of the* Mtb* P-type ATPases under stress conditions.

### 5.1. The* ctpF*,* ctpG*, and* ctpC* Genes Are Preferentially Activated in the Presence of Toxic Substances

In addition to punctual mutations in drug targets,* Mtb* possesses alternative antibiotic resistance mechanisms, such as efflux pumps, DNA repair proteins, and membrane transporters [32]. Therefore, it is of interest to know which transporters are overexpressed during the exposure of* Mtb* to toxic substances.

**Figure 2:**
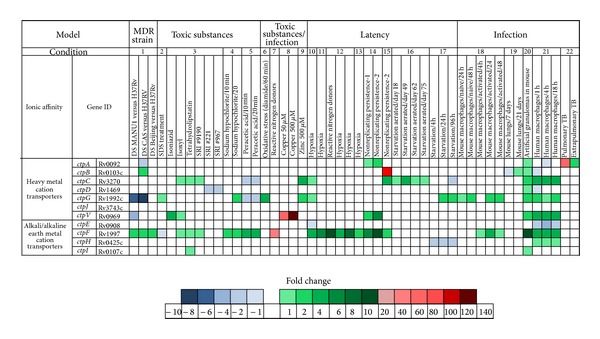
General scheme of the transcriptional behaviors of the* Mtb* P-type ATPases under stress conditions, such as toxic substances, latency, and infection. This figure was generated from the information reported in the references of Table 2 and is organized depending on the experimental conditions, which were numbered in the same table.

The CtpF and CtpC P-type ATPases are the most overexpressed transporters when* Mtb* is treated with toxic substances, including isoniazid, tetrahydrolipstatin, isoxyl, SRI #221, SRI #967, SRI #9190 [34], SDS [33], sodium hypochlorite [35], and peracetic acid [36] (Figure 2), which suggests that these transporters could contribute to the intrinsic resistance of* Mtb* to those compounds. Furthermore, CtpF, which is a possible alkali/alkaline earth metal cation transporter, is almost 60-fold overexpressed when mycobacteria face nitrogen reactive substances [38], while CtpG, a possible Zn^2+^ ATPase, is activated in response to the oxidant agent diamide [37]. As expected, the genes that respond to heavy metal poisoning (Cu^2+^ and Zn^2+^) are the genes predicted to encode heavy metal cation P-type ATPases (*ctpG*,* ctpC*, and* ctpV*) [11, 39].

### 5.2. CtpF and CtpC Are Activated under* In Vitro* Models of Hypoxia and Starvation

Nearly 95% of* Mtb* lung infections are acquired via aerosol progress to latent TB [14]. Therefore, many models of hypoxia, starvation, and murine infection have been developed to simulate the dormant state of mycobacterial latent infection, in an attempt to understand the mechanisms of* Mtb* persistence. However, these models cannot entirely represent the complexity of the granuloma [4, 48]. In the* in vitro* models of hypoxia, it has been observed that the* ctpF* gene is highly overexpressed (15 times) during low oxygen tension under microaerophilic and anaerobic conditions, suggesting a key role of the CtpF transporter in hypoxia [43, 51]. Certain heavy metal cation P-type ATPase genes, including* ctpC*,* ctpA*,* ctpV*, and* ctpB*, are also activated under hypoxic conditions, but at a lower level [13, 40, 44] (Figure 2). However,* ctpF* is the only* Mtb* P-type ATPase gene that is regulated by the global latency regulator* dosR *[43, 51]. Additionally, models that simulate nutrient starvation, a characteristic of the caseous compartments in which the tubercle bacilli reside during the latent infection (granuloma), show that the genes that code for the heavy metal pumps CtpC and CtpG are overexpressed, while CtpH is suppressed under these stress conditions [45, 46].

Alternatively, gene regulation data also show that, during the dormant phase, there is a large attenuation of the* Mtb* F_0_-F_1_-ATP synthase pump, indicating impaired ATP production during the nonreplicative state of mycobacteria [4]. A diminished ATP level would limit the overall activity of the ATPase enzymes due to the scarcity of energy sources; conversely, under this particular stress condition, P-type ATPases have been shown to be overexpressed, a biological phenomenon that highlights the importance of this type of transporters in the tubercle bacilli persistence during the latent infection. The above observations lead us to postulate that P-type ATPases could be exploited as therapeutic targets if their roles in persistence were interpreted in the opposite direction. For example, it would be possible that the activation of* Mtb* P-type ATPases during the dormant phase (using a stimulating compound) could cause a leak in the mycobacterial ATP stores, resulting in the physiological instability or death of the tubercle bacilli or forcing the bacilli to return to an active state, in which the bacteria are susceptible to common anti-TB drugs [4]. In this regard, a mycobacterial F_1_-F_0_-ATP synthase inhibitor, such as TMC207 [52], could be used in combination with compounds that mediate* Mtb* P-type ATPase activation, thus promoting ATP consumption and avoiding ATP synthesis in the pathogen.

### 5.3. The Majority of* Mycobacterium tuberculosis* P-Type ATPases Are Activated* In Vivo*

When designing new anti-TB drugs, it is important that the selected therapeutic targets play an essential role in both active disease and latent* Mtb* infections [14, 30]. Therefore, it is necessary to identify gene products that are pivotal for the persistence of* Mtb* within phagocytic cells [53]. Thus, it has been shown that the following gene products are overexpressed during infectious processes, especially during the infection of human macrophages: CtpF, CtpG, CtpV, CtpC, CtpA, CtpH, and CtpI (Figure 2). The overexpression of these genes highlights the importance of P-type ATPases in TB infection. Interestingly, the* ctpE* gene is the only gene that is downregulated during* Mtb* infection. Finally, it is worth noting that no changes were observed in the expression of the heavy metal transporter CtpJ under the stress conditions shown in Table 2. These observations strongly suggest that P-type ATPases might play essential roles during the* Mtb* infection process and that their inhibition could affect the viability and/or virulence of the tubercle bacilli.

## 6. Divergence of the* Mycobacterium tuberculosis* P-Type ATPases 

P-type ATPases share the same catalytic mechanism, independent of their origin and ion specificity [15, 19, 20]. On the other hand, to use* Mtb* P-type ATPases as therapeutic targets, it is important that differences exist between the human and mycobacterial P-type ATPases to avoid cross-reactions with the host cells [30].

### 6.1. P-Type ATPases Are Well Conserved in the* Mycobacterium tuberculosis* Complex

Preferably, a therapeutic target must be present in other pathogens belonging to the same taxonomic class, due to the fact that an inhibitor designed against a particular infectious agent may be able to act against an infection caused by any one or several of its phylogenetically related pathogens. Regarding* Mtb* P-type ATPases, it has been observed that this type of transporters is quite well conserved in the* Mtb* complex, with at least 98% identity in their amino acid sequences [22]. This high similarity may be advantageous, as the inhibitor of a particular* Mtb *P-type ATPase could be active against other members of the* Mtb* complex, which can also cause serious infectious diseases. From the experimental point of view, the fact that the attenuated and virulent* Mtb*, H37Ra, and H37Rv strains share an identical P-type ATPase sequence [22] is an advantage because it gives to the investigator the possibility to work with the attenuated and biosafe H37Ra strain, which vastly simplifies the experimental procedures oriented towards the characterization of these enzymes. Additionally, an interesting and ambitious option would be to exploit the similarities between the* Mtb* P-type ATPases to design inhibitors that can block several of these transporters. For example, the mycobacterial CtpA and CtpB transporters are predicted to be Cu^1+^P_IB-1_-type ATPases, and these enzymes share 68% identity in their amino acid sequences (see Figure S1 in Supplementary Material available online at http://dx.doi.org/10.1155/2014/296986) [22]. Thus, if an inhibitor is designed against one of these transporters, it is very likely that such an inhibitor could be active against the other enzyme.

### 6.2. Divergence between the Mycobacterial and Human P-Type ATPases

Table 1 shows that CtpE, CtpH, CtpJ, and CtpD are the most divergent* Mtb* P-type ATPases compared with their human counterparts; thus, these enzymes are the most likely to be inhibited without affecting the host cells. Conversely, CtpF, CtpV, CtpA, and CtpB are closer to human P-type ATPases, with similarities lower than 57%. This implies that, at any rate,* Mtb* P-type ATPase pumps contain a considerable proportion of divergent sequences, potential targets for inhibitor compounds. Trying to avoid possible cross-reactions of the* Mtb* P-type ATPase inhibitors with the host, the development of prodrugs, which can be converted into their active forms in the intraphagosomal environment or as products of mycobacterial metabolism, could be an interesting strategy for the discovering of new antituberculous drugs. This approach has been used successfully in other cases, including the omeprazole and thapsigargin prodrugs, which inhibit tissue-specific P-type ATPases [29].

## 7. *Mtb* P-Type ATPases as Virulence Factors

Virulence factors are involved in the invasion and persistence of pathogens inside the hosts, as well as in disease manifestations [53]. In this sense, P-type ATPases are required for the virulence of several bacterial pathogens, such as* Pseudomonas aeruginosa* and* Streptococcus pneumoniae* [27, 54–56]. Recent studies on the* Mtb* complex have considered the heavy metal cation transporters CtpC and CtpV as virulence factors whose inhibition may induce an attenuation of the tubercle bacilli [23]. However, phenotypic studies have shown that the deletion of these pumps does not result in a complete loss of virulence, suggesting the existence of compensatory mechanisms among these enzymes.

## 8. *Mycobacterium tuberculosis *P-Type ATPases in the Development of Novel Vaccines

The large number of P-type ATPases encoded in the tubercle bacillus genome suggests the evolutionary importance of* Mtb* metal cation homeostasis [17, 22]. According to this assumption, the tubercle bacillus apparently developed a compensatory strategy involving the P-type ATPases, in which the suppression of one P-type pump is counterbalanced by the overexpression of another P-type ATPase with similar activity [11]. Interestingly, it has been observed that the deletion of CtpV, one of the* Mtb* P-type ATPases, leads to an attenuation of the Δ*ctpV* mutant compared with other mycobacterial strains [17]. Then, this mechanism opens a window for the design of attenuated anti-TB vaccines and allows the construction of mycobacterial mutant strains, null in multiple P-type ATPase genes. This strategy represents a promising option for the development of a novel, live vaccine to replace the current, questionable BCG vaccine [11].

## 9. Conclusions and Outlooks

In general, most* Mtb* P-ATPases modify their expression profiles when the mycobacteria face stressful conditions, such as the presence of toxic substances, latency infection, and active disease, suggesting that these transporters may be part of the strategies used by tubercle bacilli to colonize, interact with, and/or alter the functions of the host cells. In light of these behaviors, we suggest two strategies for the rational design of* Mtb* P-type ATPases inhibitors: (i) blocking the* Mtb* P-type ATPases that are overexpressed under stress conditions, as these enzymes might be required for the persistence of the pathogen during infection and their inhibition may compromise* Mtb* viability, and/or (ii) activating the P-type ATPases during the dormant phase of* Mtb* infection to alter the strict homeostasis required by* Mtb* to survive during this process. Additionally, P-type ATPases are very well conserved among the* Mtb* complex; this may be advantageous because a potential inhibitor designed against any* Mtb* P-type ATPase could be active against other members of the* Mtb* complex, thereby combating the serious health problem caused by TB worldwide. Another remarkable feature is the considerable divergence between human and mycobacterial P-type ATPases, which makes the latter potential targets for novel anti-TB drugs. Finally, the apparent existence of compensatory mechanisms among* Mtb* P-type ATPases and the possible attenuation related to the deletion of this kind of transporters open the possibility for the design of new, anti-TB attenuated vaccines to replace the current BCG vaccine. Of course, further research must be performed to evaluate the applicability, advantages, and disadvantages of these targets, taking into account that it is essential for new drugs to be economical, as 94% of TB cases occur in extremely poor societies.

## Supplementary Material

Amino acid sequence similarities between the Mycobacterium tuberculosis P-type ATPases.

## Figures and Tables

**Table 1:** *Mtb* P-type ATPases and related human counterparts. The search for homologous ATPases was performed using the *Blastp* tool (http://blast.ncbi.nlm.nih.gov/). The table is organized according to the levels of similarity based on the “scores” from the pairwise alignment obtained using MATCHER (http://mobyle.pasteur.fr).

*Mtb* P-type ATPase	Closet human homologs		
	Pairwise alignment score	Identifier∗	Human P-type ATPase
CtpF (Rv1997)	1272	P98194	Ca-transporting ATPase
CtpV (Rv0969)	886	Q04656	Cu-transporting ATPase 1
CtpA (Rv0092)	727	P35670	Cu-transporting ATPase 2
CtpB (Rv0103c)	722	P35670	Cu-transporting ATPase 2
CtpC (Rv3270)	539	P35670	Cu-transporting ATPase 2
CtpI (Rv0107c)	450	Q13733	Na/K-transporting ATPase *α* 4
CtpG (Rv1992c)	441	P35670	Cu-transporting ATPase 2
CtpD (Rv1469)	392	P35670	Cu-transporting ATPase 2
CtpJ (Rv3743c)	377	Q04656	Cu-transporting ATPase 1
CtpH (Rv0425c)	334	Q93084	SERCA Ca ATPase
CtpE (Rv0908)	250	P98194	Ca-transporting ATPase

*UniProt identifiers (http://www.uniprot.org/).

**Table 2:** Studies investigating *M. tuberculosis* gene expression profiles under stress conditions.

Model	Experimental conditions		Methodology∗∗	References
MDR strains	1	Comparison between MDR and H37Rv strains	MA	Chatterjee et al. (2013) [32]

Toxic substances (*in vitro*)	2	SDS	RT-qPCR and MA	Manganelli et al. (2001) [33]
	3	Isoniazid, isoxyl, tetrahydrolipstatin, SRI #221, SRI #967, and SRI #9190	MA	Waddell et al. (2004) [34]
	4	Sodium hypochlorite	MA	Jang et al. (2009) [35]
	5	Peracetic acid	MA	Nde et al., (2011) [36]

Toxic substances related to infection (*in vitro*)	6	Oxidative stress	MA	Manganelli et al. (2002) [37]
	7	Nitrogen reactive substances	RT-qPCR and MA	Ohno et al. (2003) [38]
	8	Physiological levels of copper	RT-qPCR	Ward et al. (2008) [39]
	9	Physiological levels of zinc	RT-qPCR and MA	Botella et al. (2011) [11]

Latency (*in vitro*)	10	Hypoxia	MA	Sherman et al. (2001) [40]
	11	Hypoxia	MA	Bacon et al. (2004) [41]
	12	Hypoxia and nitric oxide	MA	Voskuil et al. (2003) [42]
	13	Steady culture	RT-qPCR and MA	Kendall et al. (2004) [43]
	14	Nonreplicating persistence (NRP)	MA	Muttucumaru et al. (2004) [44]
	15	Nonreplicating persistence (NRP)	Proteomic	Cho et al. (2006) [13]
	16	Starvation	MA	Hampshire et al. (2004) [45]
	17	Starvation	MA	Betts et al. (2002) [46]

Infection (*in vivo*)	18	Mouse macrophage infection	MA	Schnappinger et al. (2003) [47]
	19	Mouse lung infection	MA	Talaat et al. (2004) [31]
	20	Artificial granulomas in mice	MA	Karakousis et al. (2004) [48]
	21	Human dendritic cells and macrophage infection	MA	Tailleux et al. (2008) [49]
	22	Human lung infection	RT-qPCR	Kumar et al. (2011) [50]

** MA, microarrays.
